# Metabolic and Vascular Inflammation in Alopecia Areata: Linking Uric Acid, Lipid Imbalance and ICAM‐1 Upregulation

**DOI:** 10.1111/exd.70186

**Published:** 2025-12-12

**Authors:** Madoc Dawson, Derek Pye, Rebecca Mahon, George Taylor, Asim Shahmalak, Bessam Farjo, Nilofer Farjo, Matthew Harries, Talveen S. Purba

**Affiliations:** ^1^ Centre for Dermatology Research, Division of Musculoskeletal and Dermatological Sciences, School of Biosciences, Faculty of Biology, Medicine, and Health The University of Manchester, Manchester Academic Health Science Centre Manchester UK; ^2^ Biological Mass Spectrometry Facility The University of Manchester Manchester UK; ^3^ Crown Clinic Manchester UK; ^4^ Farjo Hair Institute Manchester UK; ^5^ Salford Royal Hospital, Northern Care Alliance NHS Foundation Trust Manchester Academic Health Science Centre Manchester UK

## Abstract

Alopecia areata (AA) is an inflammatory hair loss disorder caused by an immune‐mediated attack of the hair follicle (HF) bulb. Active disease is characterised by a peribulbar proinflammatory infiltrate, HF immune privilege collapse and premature catagen induction, yet the underlying drivers of AA remain poorly understood. With comparable autoimmune inflammatory conditions displaying metabolic alterations, we hypothesised that AA is marked by similar pathobiological changes. To investigate this, we utilised an exploratory metabolomics‐based discovery liquid chromatography mass spectrometry (LC–MS) approach. This yielded 32 putatively annotated metabolites significantly altered between lesional and nonlesional AA scalp. Notably, 13‐HODE, a linoleic acid metabolite linked to vascular function, was decreased, whilst uric acid (UA), a purine degradation metabolite linked to vascular dysfunction, was increased in the lesional scalp. Moreover, serum LC–MS revealed elevated UA in AA compared to controls, which is linked to systemic endothelial dysfunction. CD31+/ICAM‐1+ immunofluorescence co‐expression analysis revealed elevated vascular inflammation and endothelial cell activation in the AA scalp. We also experimentally provoked the same response in ex vivo human HF culture via UA or fructose (which increases UA) supplementation. Interestingly, the fructose‐generating polyol pathway enzymes, AKR1B1 and SORD, are expressed in the HF, with significantly increased AKR1B1 immunoreactivity in lesional AA HFs, suggesting that fructose can be locally generated by the HF and may contribute to elevated UA levels in AA. Together, these metabolic changes point towards UA‐linked microvascular dysfunction in AA, inviting exploration of whether strategies to improve endothelial function and regulate UA are effective in managing AA.

## 1 Introduction

Alopecia areata (AA) is a nonscarring, immune‐mediated hair loss disorder with an estimated UK point prevalence of 0.58% in 2018 and a peak incidence age range of 25–29 years [1]. It is the second most diagnosed form of hair loss, accounting for 18% of hair loss diagnoses [2]. AA typically presents as patchy hair loss on the scalp, but it can also progress to total scalp hair loss (alopecia totalis (AT)) or complete scalp and body hair loss (alopecia universalis (AU)) [3].

AA is a T‐cell‐mediated autoimmune condition impacted by both genetic and environmental factors, inducing the collapse of anagen hair follicle (HF) immune privilege (IP), premature catagen induction and HF dystrophy [4, 5, 6]. The collapse of IP results in a loss of HF‐protective immunosuppressive mechanisms driven by an increase in pro‐inflammatory cytokines, manifesting with increased major histocompatibility complex (MHC) class I and II expression in the proximal HF epithelium [7]. As a result, CD4+ T‐helper cells and CD8+ cytotoxic T‐cells infiltrate in and around the HF bulb [8], often alongside other immunocytes such as natural killer (NK) cells, γδ T cells, mast cells and macrophages [9].

The role of systemic inflammation on metabolism and cardiovascular risk is a growing area of research. Various immune‐mediated inflammatory diseases (IMID), including rheumatoid arthritis, psoriasis and systemic lupus erythematosus, show an increased risk of metabolic syndrome as well as notable changes in immunometabolism [10, 11, 12]. This may also be the case for AA, especially with higher rates of metabolic syndrome being reported [13]. However, despite being central to hair growth [14, 15], metabolism has been underexplored in AA versus comparable IMIDs; diseases that share several common risk factors, including shared genetic risk loci [6], increased rates of comorbid autoimmune diseases, systemic inflammation and heightened cardiovascular risk and endothelial dysfunction [16, 17].

To address this gap, we conducted exploratory Liquid Chromatography Mass Spectrometry (LC–MS) using AA tissue samples, demonstrating localised metabolic shifts, particularly in lipid and purine metabolism (i.e., changes in 13‐HODE and uric acid (UA)). Given that comparable IMIDs and metabolic disturbances have been linked to microvascular changes [18, 19], we then link this to heightened ICAM‐1 expression within the lesional AA scalp endothelium as a novel in situ finding. We further show that increased ICAM‐1 expression can be independently induced in an ex vivo scalp HF organ culture model through exposure to UA or fructose (which itself contributes to UA generation [20, 21]) and that the HF may locally produce fructose via the polyol pathway, suggesting an endogenous source for metabolic‐vascular stress in AA.

## 2 Methods

### 2.1 Tissue Preparation—AA And Whole Control Scalp Biopsies

The AA diagnosis was established clinically by a clinician experienced in hair loss management (MH). Nonlesional and lesional scalp tissue biopsies (6 mm punch), from Salford Royal Hospital, Salford, UK, were obtained from AA patients following informed consent, in accordance with ethics approval from the West Midlands–South Birmingham Research Ethics Committee. Lesional scalp biopsies were taken from sites at the margin of an existing site of hair loss, and nonlesional biopsies were taken from neighbouring scalp sites that, where possible, showed no clinically visible indications of active hair loss. If patients had previously received intralesional steroid therapy, biopsies were only taken with a minimum washout period of 3 months since the most recent treatment. The patient sample group is described in Table 1. One patient presented with a positive pull test throughout the scalp, which may indicate lesional activity—analyses in which this material was used were performed, including and excluding data from this patient, with no impact on overall trends or conclusions (Figure 2, Figure S1). Biopsies for LC–MS were directly flash frozen in liquid nitrogen and stored at −80°C. Biopsies for immunofluorescence were embedded in optimal cutting temperature compound (OCT), frozen in liquid nitrogen, and stored at −80°C. Excess occipital scalp from strip surgeries was provided by Crown Cosma Clinic and The Farjo Hair Institute, as well as control scalp biopsies being obtained commercially from Caltag Medsystems.

**TABLE 1:** Table describing key features of all participants with alopecia areata whose serum or scalp biopsies were used to produce the results detailed in this manuscript.

Age	Sex	SALT	Hair loss treatments	Age of onset	Signs of active hair loss	Sample type
21	Female	80	Betnovate	13	N/A	Serum
48	Female	15	Dermovate Prior ILT (> 6 months)	46	Exclamation mark hairs, positive pull test	Serum
41	Female	28	Prior ILT (> 3 months)	N/A	N/A	Serum
28	Male	78	Elocon	13	N/A	Serum/Biopsy
52	Male	62	Dithrocream	48	N/A	Serum/Biopsy
46	Female	76.4	Cyclosporin (tapering to stop)	45	Exclamation mark hairs, positive pull test throughout scalp	Serum/Biopsy
42	Female	4	Prior ILT (> 3 months)	17	Exclamation mark hairs	Serum/Biopsy
46	Female	15	None	44	N/A	Serum
52	Female	55.6	None	10	N/A	Serum
38	Female	8.3	None	38	N/A	Serum
25	Female	27.4	Minoxidil	15	Exclamation mark hairs, positive pull test	Serum
39	Female	N/A	None	23	N/A	Serum/Biopsy
33	Female	< 5	Previous ILT (> 3 months, biopsy site left untreated)	21	Exclamation mark hairs	Serum/Biopsy
56	Male	3	Minoxidil, ILT (eyebrows only, biopsy site untreated), Bitamoprost	53	N/A	Serum/Biopsy
23	Male	5.5	Minoxidil	20	N/A	Serum
21	Female	40	None	7	Exclamation mark hairs	Serum
33	Female	N/A	None	31	N/A	Serum
35	Male	47	None	20	N/A	Serum
30	Female	90	None	Teens	N/A	Serum
32	Female	71	None	9	N/A	Serum
29	Female	N/A	None	23	N/A	Serum

*Note:* This includes age, sex, SALT score as a severity measure, any treatments for hair loss that the patient was taking at the time of biopsy, age of onset (i.e., first experienced symptoms), clinical observations that suggest active hair loss is ongoing, and the sample types used. One patient included had a positive pull test across the scalp. All analyses that included this patient have been repeated and included in Figure S1 in case this is an indication that there was not a truly nonlesional site for biopsy. Abbreviation: ILT, intralesional triamcinolone.

For LC–MS, the Covaris CryoPrep was employed. Samples were pulverised using the Covaris cryoPREP CP02 and resuspended in 1:1 water/methanol using a vortex mixer. This suspension was shaken overnight at 1°C in a Thermomixer at 2000RPM. Samples were then centrifuged in a prechilled centrifuge at 20 000 × g for 3 min. The supernatant was transferred to a new microfuge tube and stored at −20°C.

For immunofluorescence, 7 μm frozen tissue sections were prepared on Superfrost Plus slides (ThermoFisher Scientific, Waltham, MA).

### 2.2 Sample Preparation—Serum

Diagnosis, consent and ethics approval for serum acquisition were as described in the scalp tissue preparation section above. Blood samples were collected in 3.5 –5 mL gold top BD Vacutainers with separator gel plugs (product code 367956/367954) before centrifugation at 3000 rpm for 12 min. The serum was removed by pipetting and stored at −80°C. The patient sample group is described in Table 1 (average age 37, 74% female, 26% male). Healthy control serum was obtained commercially from BioIVT (average age 38, 77% female, 23% male).

### 2.3 Tissue Preparation—Microdissected Occipital Scalp Human Hair Follicles

Extracted follicular units from the occipital scalp were obtained from healthy patients undergoing HF transplantation following informed consent. These were provided by Crown Cosma Clinic, Manchester, UK and The Farjo Hair Institute, Manchester, UK.

HF were microdissected and allowed to rest at 4°C overnight. The follicles were then placed individually into wells with standard Hair Follicle Organ Culture Media (HFOC) (Williams E medium, 100 U/mL penicillin, 100 μg/mL streptomycin, 2 mM l‐glutamine, 10 μg/mL insulin and 10 ng/mL hydrocortisone [22]) at 37.5°C for 48 or 72 h with a media change on day 2 during 72 h cultures. The control was HFOC media as described above. For uric acid cultures, HFOC media was supplemented with 200 μM or 1000 μM uric acid. Fructose/glucose cultures were HFOC media supplemented with 10 mM or 40 mM glucose or fructose for a total of five conditions. Afterwards, HFs were embedded in OCT, frozen in liquid nitrogen and stored at −80°C.

### 2.4 Liquid Chromatography Mass Spectrometry

LC–MS was performed by the University of Manchester BioMS facility and analysed using a SCIEX Exion LC system consisting of two AD high‐pressure gradient pumps, vacuum degasser, solvent valve, AC column oven and AC Autosampler, coupled to a SCIEX 7600 ZenoTOF Q‐TOF mass spectrometer with TurboV Optiflow ion source running a 50 μm ESI probe. The system was controlled by SCIEX OS v3.0.

#### 2.4.1 Tissue Sample Preparation

Samples from Covaris CryoPrep (see tissue preparation section) were centrifuged at 20 000 × g for 3 min. An aliquot of 500 μL was taken to dryness in a SpeedVac centrifuge. The sample was then reconstituted in 100 μL acetonitrile/water 5:1. Quality control samples were made by pooling 10 μL from each sample.

#### 2.4.2 Serum Sample Preparation

To a 100 μL serum sample, 300 μL acetonitrile was added and centrifuged at 20 000 × g for 3 min at 4°C to precipitate endogenous proteins. The resulting supernatant was applied to a phospholipid depletion plate (Biotage) under 3 psi positive pressure for 10 min. The resulting filtrate was transferred to a glass autosampler vial with a 300 μL insert and dried under vacuum. The sample was resuspended in 100 μL 5:1 acetonitrile/water and capped.

A sample volume of 10 μL (CryoPrep) or 5 μL (serum) was injected onto a 50 μL sample loop with or injection needle wash, the same as the mobile phase start conditions. The injection cycle time was 1 min per sample. Separations were performed using an Agilent Poroshell 120 HILIC‐Z column with dimensions of 150 mm length, 2.1 mm diameter and 2.7 μm particle size equipped with a guard column of the same phase. Mobile phase A was water with 10 mM ammonium acetate adjusted to pH 9 with ammonium hydroxide and 20 μM medronic acid, mobile phase B was 85:15 acetonitrile and water with 10 mM ammonium acetate adjusted to pH 9 with ammonium hydroxide and 20 μM medronic acid. Separation was performed by gradient chromatography at a flow rate of 0.25 mL/min, starting at 96% B for 2 min, ramping to 65% B over 20 min, holding at 65% B for 2 min, then back to 96% B. Re‐equilibration time was 5 min. Total run time, including 1 min injection cycle, was 30 min.

The mass spectrometer was run in negative mode under the following source conditions: ion spray voltage, −4500 V; curtain gas pressure, 50 psi; temperature, 400°C; ESI nebuliser gas pressure, 50 psi; heater gas pressure, 70 psi; declustering potential, −80 V.

For metabolomics, data were acquired in an information‐dependent manner across 10 product ion scans, each with an accumulation time of 100 ms and a TOF survey scan with an accumulation time of 250 ms. Zeno pulsing was used for both scan types. Total cycle time was 1.3 s. Collision energy was determined using the formula CE (V) = 0.084 × *m/z* + 12 up to a maximum of 55 V. Isotopes within 4 Da were excluded from the scan.

Acquired metabolomics data were checked in PeakView 2.2 and imported into Progenesis Qi 3.0 for Metabolomics, where they were aligned, peaks were picked, normalised to all compounds, and deconvoluted according to standard Progenesis workflows. Annotations were made by searching the accurate mass, MS/MS spectrum and isotope distribution ratios of acquired data against the National Institute of Standards and Technology (NIST) MS/MS metabolite library. Metabolites were identified by searching retention times and accurate masses against an in‐house chemical standard library.

For serum analysis, data were acquired in a data‐independent manner using SWATH in the range of 50–1000 m/z, split across 78 variable‐size windows (79 experiments including TOF survey scan), each with an accumulation time of 20 ms. Total cycle time was 1.66 s. The collision energy of each SWATH window was determined using the formula CE (V) = 0.084 × m/z + 12 up to a maximum of 55 V. The table showing the m/z ranges and respective collision energies for relevant SWATH windows is given in Table 2.

**TABLE 2:** Table showing the *m/z* ranges and respective collision energies for SWATH windows utilised for serum LC–MS of fructose, glucose and uric acid.

Compound	Fructose	Glucose	Uric acid
Retention time (min)	3.26	4.5	4.5
SWATH experiment	37	37	25
Collision energy (V)	29	29	25
Precursor ion m/z	215.0328	215.0328	167.0214
Quantifier product ion m/z	70.9731	70.9731	123.9653
Qualifier product ion m/z 1	88.9735	88.9735	96.0205
Qualifier product ion m/z 2	161.8419	161.8419	167.0214

Acquired serum data were processed in MultiQuant 3.0.2. Peaks from MS1 and MS2 data were picked and matched against a metabolite library of 235 standards, based on retention time and mass error of ±0.025 Da. Data exported from MultiQuant 3.0.2 were further sorted, filtered and scored using a custom VBA macro in Excel, based on presence, peak area and coelution of precursor and fragment ions.

Data visualisation was performed using GraphPad Prism 9 (GraphPad Software). Pathway enrichment analysis was performed using MetaboAnalyst 6.0 and the Small Molecule Pathway Database (SMPDB) pathway library.

### 2.5 Immunofluorescence Staining and Microscopy

CD31 (1:50, Dako, product code M0823), ICAM‐1 (1:100, Abcam, product code AB109361), AKR1B1 (1:100, Abcam, product code AB268058, permeabilised with 0.5% Triton X‐100, blocked with normal goat serum), SORD (1:100, Abcam, product code AB189248) and GLUT5 (1:100, Alomone, product code AGT‐025) followed the same immunofluorescence protocol, except where previously indicated. Samples were fixed in ice‐cold acetone (−20°C) before washing (3 × 5 min) with phosphate‐buffered saline (PBS). Samples were then blocked in SuperBlock Blocking Buffer (ThermoFisher Scientific, Waltham, MA) for 45 min at RT before washing again. Primary antibodies were incubated overnight at 4°C. Secondary Alexa Fluor antibodies were utilised at 1:200 as appropriate (goat anti‐mouse 488/594 and goat‐anti rabbit 488/594) (ThermoFisher Scientific, product codes #A11001, #A11005, #A11008, #A11037) before washing. Nuclear stain Hoechst 33342 (1:1000, 10 mg/mL stock, ThermoFisher) was applied for 5 min at RT before washing again (3 × 2 min).

Images were acquired using the Keyence BZ‐8000 fluorescent microscope, with the exception of Figures 3A and 4E, which were acquired using the Keyence BZ‐X810. Single‐channel fluorescent images were analysed in ImageJ software (NIH) and QuPath [23]. QuPath was used for automated cell detection to generate a mask for cell counting analyses. Counting of positive/double positive cells was performed manually and blinded. Perifollicular CD31/ICAM‐1 double‐positive cells were quantified within 100 μm of the HF connective tissue sheath (CTS). CD31/ICAM‐1 double‐positive cells within the papillary dermis were quantified within 300 μm of the proximal border of the epidermis and at least 100 μm away from HFs. ICAM‐1 expression was considered exclusively within cells positive for CD31. Values were graphed and statistically analysed using GraphPad Prism 9 (GraphPad Software). One‐way ANOVA was used for all analyses, followed by either Dunnett's multiple comparison test when comparing all conditions to the control, or Tukey's multiple comparison test in instances where all conditions are compared to all other conditions. Colour blind friendly pseudocoloured versions of dual‐stain fluorescence images from Figures 2 and 3 are provided in Figures S1 and S2, respectively.

### 2.6 In Situ Hybridisation

RNA fluorescent in situ hybridisation was performed using RNAscope RNAscope Multiplex Fluorescent V2 Assay (Advanced Cell Diagnostics, product code #323270) and probes Hs‐SORD‐O1‐C1 (Advanced Cell Diagnostics, product code #1274831‐C1) and Hs‐AKR1B1 (Advanced Cell Diagnostics, product code #812951).

## 3 Results

### 3.1 Exploratory Metabolomics Reveals Uric Acid and 13‐HODE Levels Are Altered in Lesional Versus Nonlesional Alopecia Areata Scalp

**FIGURE 1:**
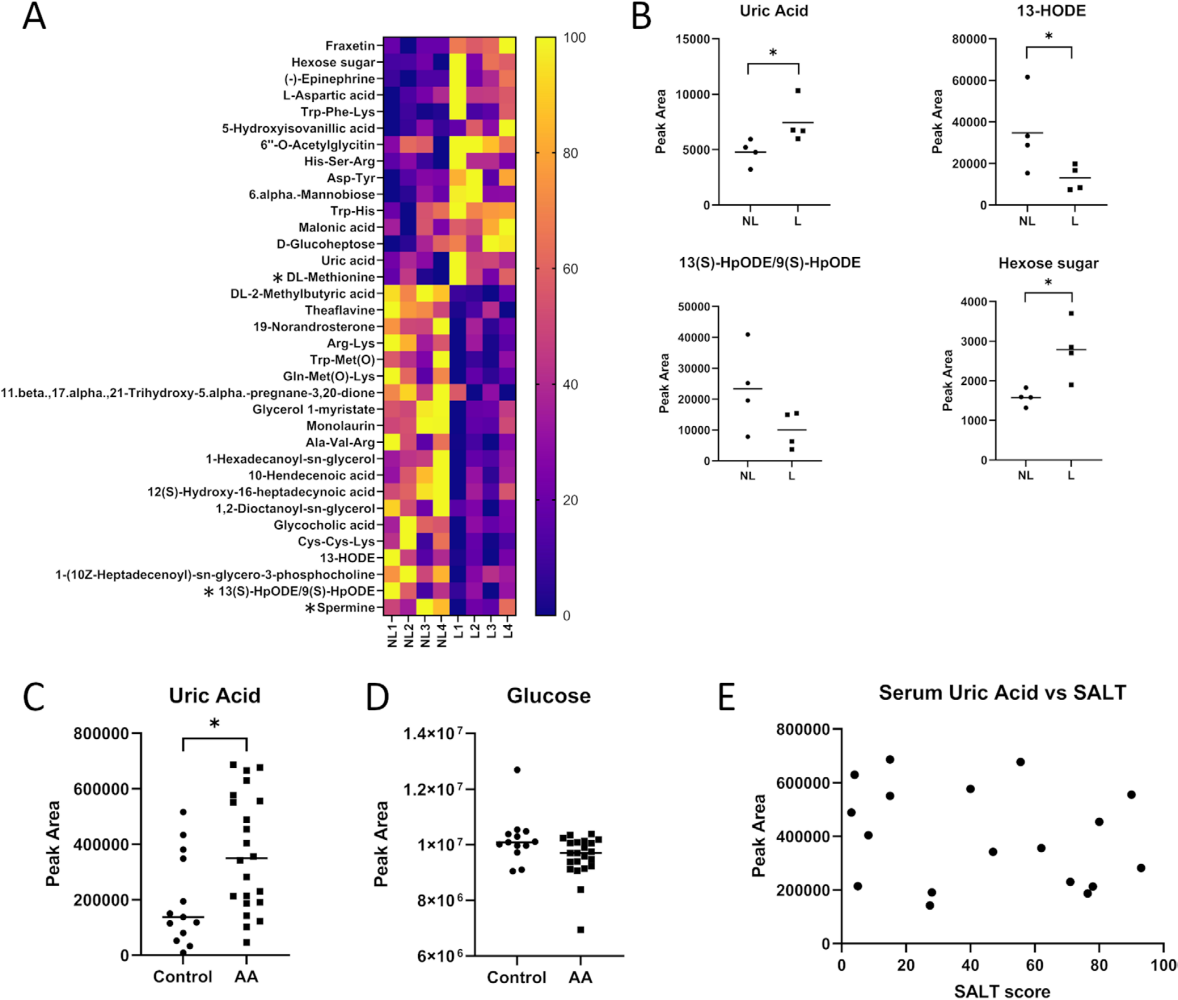
(A) Heatmap of putatively annotated metabolites of interest detected by LC–MS found to be altered (one‐way ANOVA, *p* = < 0.05) between nonlesional (NL) and lesional (L) AA scalp (*n* = 4). Metabolites marked with * are those that were not statistically significant. (B) Column scatter plots of LC–MS data highlighting specific altered metabolites of interest from the data shown in (A). *p = < 0.05. (C) Column scatter plot of LC–MS serum uric acid peak area between AA and control serum (*n* = 21 AA, 13 control). *p = < 0.05. Line at the median. (D) Column scatter plot of LC–MS serum glucose peak area between AA and control serum (*n* = 21 AA, 13 control). Line at the median. (E) Correlation analysis plot showing no correlation of LC–MS serum uric acid peak area and SALT score.

Using LC–MS on lesional and nonlesional AA scalp samples (*n* = 4), we putatively annotated 827 metabolites (inclusive of key metabolites involved in central carbon metabolism and amino acid metabolism) with high confidence based on internal LC–MS accuracy criteria. Of these, 32 metabolites of interest were significantly altered (one‐way ANOVA, *p* = < 0.05) between lesional and nonlesional scalp skin (Figure 1A, Table S1). Unbiased pathway enrichment analysis (MetaboAnalyst 6.0, SMPDB pathway library) identified no significantly enriched pathways (*p* = < 0.05). Therefore, within this study, we opted for a biased preselection of metabolites with reported relevance to autoimmune or inflammatory conditions in the literature [24, 25, 26, 27].

Amongst these, uric acid (UA) was significantly increased. Additionally, we show a trending but nonsignificant decrease in a transient linoleic acid metabolite annotated as either 13‐(S)‐HpODE or 9‐(S)‐HpODE (which isomer was detected could not be confidently distinguished). The downstream product of 13‐(S)‐HpODE, 13‐HODE [28], was significantly decreased in lesional AA scalp (Figure 1B).

We further identified a significant increase in a hexose sugar in lesional AA samples, originally putatively annotated as fructose. However, several other hexose sugars scored very similarly (including but not limited to glucose, galactose and mannose), leading us to downgrade this to a level 3 putatively characterised compound class [29].

Methionine, an important precursor to S‐adenosylmethionine (SAM), which mediates antioxidant defence, immune regulation and polyamine biosynthesis [30], showed an increase in lesional AA bordering on significance (*p* = 0.059071) (Figure 1A). This was coupled with a trending decrease in the polyamine spermine (*p* = 0.073) (Figure 1A), suggesting that methionine metabolism may also be disrupted in AA.

Together, these data provide the first insights into the local metabolic changes seen in the affected scalp from patients with active AA.

### 3.2 Serum Uric Acid Levels are Increased in Patients With Alopecia Areata

As increased UA is associated with systemic inflammation and endothelial dysfunction, and changes were seen in UA levels in lesional AA scalp, we next considered whether systemic UA levels were increased in AA patients compared to healthy controls. Relative quantitation of LC–MS serum analysis showed a significant increase in UA levels in the serum of AA patients (*n* = 21, mean = 369 070) compared to healthy controls (*n* = 13, mean = 197 743) (Figure 1C). This shows that elevated serum UA levels in AA patients are consistent with local changes in lesional AA tissue and are indicative of broader systemic inflammation, though without donor metabolic profiles, we cannot rule out secondary metabolic changes between groups, as opposed to primary AA‐related changes.

Additional statistical analysis was performed to assess whether there was any correlation between AA severity, as measured by SALT score, and serum UA levels. Pearson correlation analysis showed no correlation between the severity of AA and serum UA (Figure 1E).

Using the same method, we next measured glucose and fructose levels of the same cohorts. Fructose levels were too low to be detectable by this technique in these samples. However, glucose levels were found to be unchanged between AA and control serum (Figure 1D). Whilst this shows a lack of systemic changes in serum glucose, this does not preclude local tissue‐specific (i.e., lesional AA) metabolic disruption that influences the levels of individual, as yet unidentified, hexose sugars.

### 3.3 Alopecia Areata Scalp Skin Shows Increased Expression of the Endothelial Activation Marker ICAM‐1 in CD31+ Cells

High UA has been linked to endothelial damage and inflammation [31], and decreased 13‐HODE is seen in response to endothelial damage to allow increased platelet adhesion [32, 33]. To investigate potential HF‐associated endothelial damage, we used the endothelial cell marker, CD31 and we analysed ICAM‐1 expression, a marker of endothelial activation [34], within endothelial cells [35] via immunofluorescence on healthy control, nonlesional and lesional AA scalp skin tissue sections.

**FIGURE 2:**
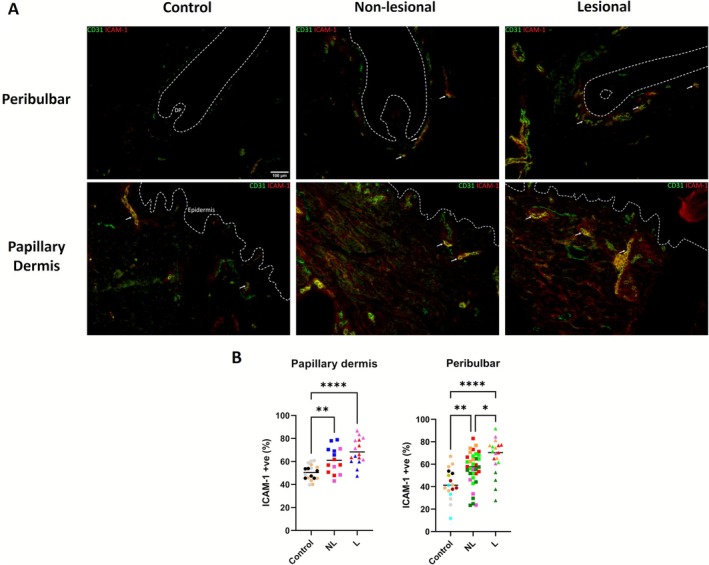
(A) Representative immunofluorescence microscopy of CD31 and ICAM‐1 dual stains in healthy control, nonlesional AA and lesional AA scalp. Hoechst channel removed to improve legibility (Originals and pseudocoloured colour blind friendly versions available in Figure S1). (B) Column scatter plots of dual‐positive CD31+/ICAM‐1+ cells as a percentage of all CD31+ cells in the peribulbar region and papillary dermis of control, nonlesional AA and lesional AA scalp (*n* = 5, 2–4 HFs per patient per condition) (one‐way ANOVA, Dunnett's multiple comparisons test, *p* = < 0.05). *p = < 0.05; **p = < 0.01; ****p = < 0.0001. Line at the median. Data points of the same colour represent data from the same HF donor.

The percentage of CD31+ endothelial cells co‐expressing ICAM‐1 in nonlesional and lesional AA scalp tissue sections was increased compared to controls (Figure 2), with this increase trending higher at lesional AA sites. This increase was apparent in regions proximal to the HF bulb (i.e., the site of immune attack in AA) as well as within the papillary dermis, a highly vascularised region of dermis distant from the anagen HF bulb (Figure 2), suggesting that endothelial activation in AA is not limited to the perifollicular regions.

### 3.4 Uric Acid and Fructose Increase the Number of Perifollicular ICAM‐1+/CD31+ Endothelial Cells in Human Hair Follicles Ex Vivo

**FIGURE 3:**
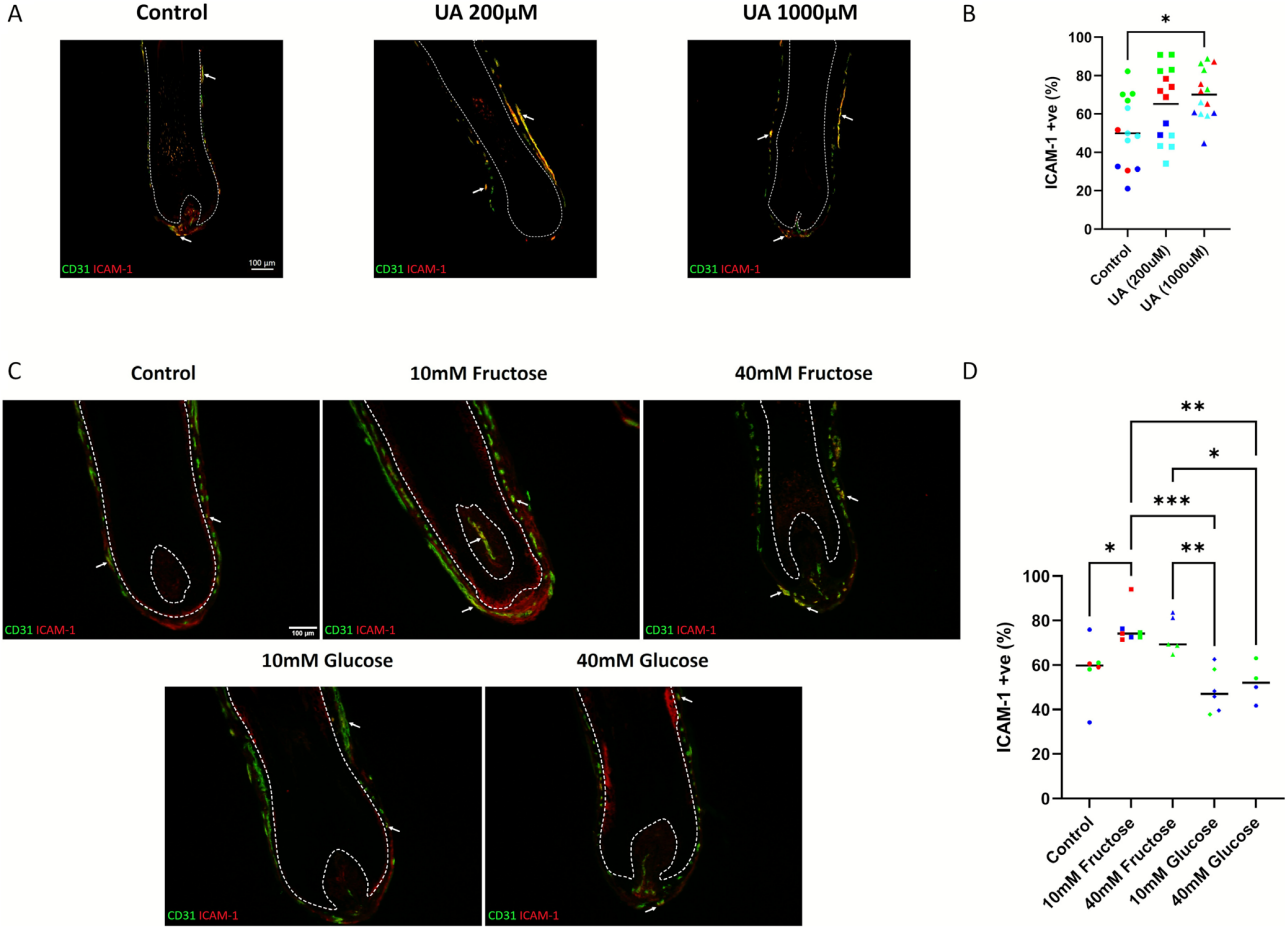
(A) Representative immunofluorescence microscopy of CD31 and ICAM‐1 dual stains in human hair follicles cultured with uric acid (UA) for 72 h (control, 200 μM, 1000uM). Hoechst channel removed to improve legibility (Originals and pseudocoloured colour blind friendly versions available in Figure S2). (B) Column scatter plots of dual‐positive CD31+/ICAM‐1+ cells as a percentage of all CD31+ cells in human HFs cultured with uric acid (UA) for 72 h (control, 200 μM, 1000 μM) (*n* = 3, 2–4 HFs per patient per condition) (one‐way ANOVA, Tukey's multiple comparisons test, *p* = < 0.05). *p = < 0.05 (all conditions compared to all other conditions; nonsignificant results not shown on graph). Line at the median. Data points of the same colour represent data from the same HF donor. (C) Representative immunofluorescence microscopy of CD31 and ICAM‐1 dual stains in human hair follicles cultured with supplemental fructose or glucose for 72 h (control, 10 mM fructose/glucose, 40 mM fructose/glucose). Hoechst channel removed to improve legibility (Originals and pseudocoloured colour blind friendly versions available in Figure S2). (D) Column scatter plots of dual‐positive CD31+/ICAM‐1+ cells as a percentage of all CD31+ cells in human HFs cultured with supplemental fructose or glucose for 72 h (control, 10 mM fructose/glucose, 40 mM fructose/glucose). (control/10 mM fructose *n* = 3, 10 mM glucose and 40 mM fructose/glucose *n* = 2, 2–4 HFs per patient per condition) (one‐way ANOVA, Tukey's multiple comparisons test, *p* = < 0.05). *p = < 0.05; **p = < 0.01; ***p = < 0.001 (all conditions compared to all other conditions; nonsignificant results not shown on graph). Line at the median. Data points of the same colour represent data from the same HF donor.

Next, to examine a potential link between increased UA levels and endothelial activation in AA, we treated healthy occipital scalp anagen HFs ex vivo with UA for 72 h and subsequently quantified HF‐associated CD31/ICAM‐1 double‐positive cells. This showed a significant increase in the percentage of CD31/ICAM‐1 double‐positive cells in the peribulbar region of HFs treated with 1000 μM UA compared to control, with a trending increase in the 200 μM UA condition (Figure 3A,B). These data show that UA can increase the expression of ICAM‐1 in CD31+ cells in human HFs ex vivo, supporting a link between increased UA and endothelial activation as a novel pathogenic mechanism in AA.

We next treated human HFs with fructose, a metabolic‐inflammatory stressor [36, 37] that contributes to the generation of UA via the purine degradation pathway [20, 38], as well as with glucose, another potential annotation for the hexose sugar that is linked to metabolic syndrome, diabetes and cardiovascular disease [39].

HFs cultured with fructose for 72 h showed a significant increase in the percentage of CD31/ICAM‐1 double‐positive cells in the peribulbar region of HFs treated with 10 mM fructose (Figure 3C,D). Forty millimolar fructose treatment also showed a trending, albeit nonsignificant, increase in CD31/ICAM‐1 double‐positive expression (Figure 3C,D). Emphasising the unique properties of fructose in provoking ICAM‐1 expression, CD31/ICAM‐1 double‐positive expression in HFs cultured with 10 mM and 40 mM glucose remained unchanged compared to untreated control HFs (Figure 3C,D). This also rules out the possibility of these ICAM‐1 changes being a result of a change in osmolarity caused by fructose supplementation. These findings support that fructose is independently capable of inducing the expression of the ICAM‐1 in CD31+ cells in human HFs ex vivo. Considered alongside the altered UA levels and UA culture results, this indicates a link between increased fructose and endothelial activation as a novel pathogenic mechanism in AA.

### 3.5 Human Hair Follicles Express the Polyol Pathway Proteins AKR1B1 and SORD, With AKR1B1 Expression Being Increased in Alopecia Areata

Previous research shows that UA, in particular hyperuricaemia, upregulates the fructose‐generating polyol pathway in various tissues, including human vascular endothelial cells, resulting in more glucose being converted to fructose [21, 40, 41]. Fructose metabolism also results in increased UA through the production of AMP, which is converted to IMP and subjected to the purine degradation pathway [20, 38]. Due to the specific local changes in UA in lesional AA scalp, we hypothesised that human scalp skin HFs can produce fructose endogenously, similar to the liver, kidney and brain [42], and that this may be altered in AA. Therefore, we studied the expression of *SORD* and *AKR1B1*, two critical enzymatic components of the polyol pathway [42] in the bulb, using RNAScope in situ hybridisation.

**FIGURE 4:**
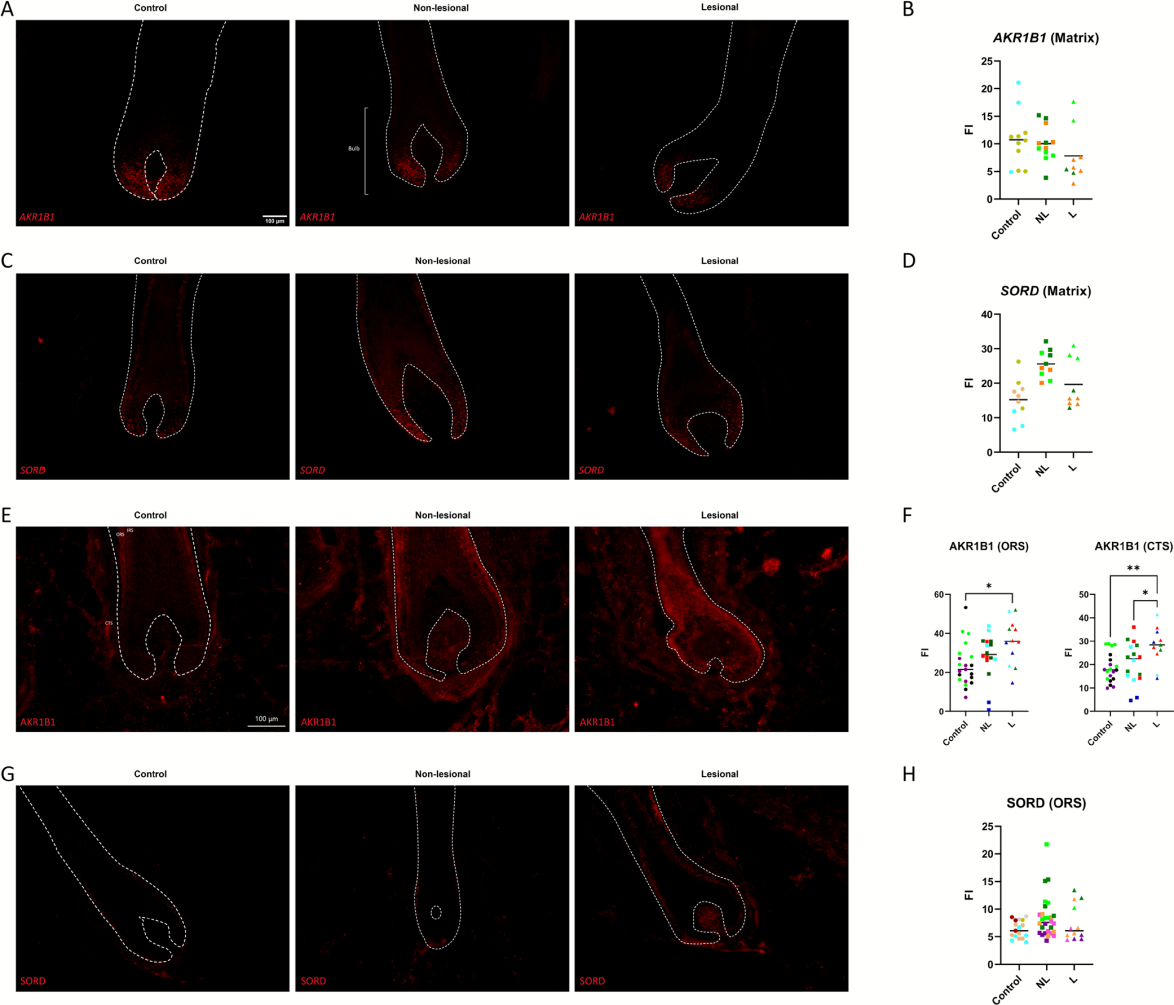
(A) Representative immunofluorescence microscopy of *AKR1B1* RNAScope in healthy control, nonlesional AA and lesional AA scalp. Hoechst channel removed to improve legibility (Originals available in Figure S3). (B) Column scatter plots of bulbar matrix *AKR1B1* fluorescence intensity (FI) in healthy control, nonlesional AA and lesional AA scalp (control *n* = 2, NL/L *n* = 3) (one‐way ANOVA, Tukey's multiple comparisons test, *p* = < 0.05). Line at the median. Data points of the same colour represent data from the same donor. (C) Representative immunofluorescence microscopy of *SORD* RNAScope in healthy control, nonlesional AA and lesional AA scalp. Hoechst channel removed to improve legibility (Originals available in Figure S3). (D) Column scatter plots of bulbar matrix *SORD* fluorescence intensity (FI) in healthy control, nonlesional AA and lesional AA scalp (*n* = 3) (one‐way ANOVA, Tukey's multiple comparisons test, no significant differences, *p* = >0.05) (all conditions compared to all other conditions; nonsignificant results not shown on graph). Line at the median. Data points of the same colour represent data from the same donor. (E) Representative immunofluorescence microscopy of AKR1B1 immunohistochemistry in healthy control, nonlesional AA and lesional AA scalp. Hoechst channel removed to improve legibility (Originals available in Figure S3). (F) Column scatter plots of outer root sheath (ORS) and connective tissue sheath (CTS) fluorescence intensity (FI) in healthy control, nonlesional AA and lesional AA scalp (control *n* = 3, NL/L *n* = 4) (one‐way ANOVA, Tukey's multiple comparisons test, *p* = > 0.05) (all conditions compared to all other conditions; nonsignificant results not shown on graph) *p = < 0.05; **p = < 0.01. Line at the median. Data points of the same colour represent data from the same donor. (G) Representative immunofluorescence microscopy of SORD immunohistochemistry in healthy control, nonlesional AA and lesional AA scalp. Hoechst channel removed to improve legibility (Originals available in Figure S3). (H) Column scatter plots of SORD outer root sheath (ORS) fluorescence intensity (FI) in healthy control, nonlesional AA and lesional AA scalp (control *n* = 4, NL/L *n* = 5) (one‐way ANOVA, Tukey's multiple comparisons test, no significant differences, *p* = > 0.05) (all conditions compared to all other conditions; nonsignificant results not shown on graph). Line at the median. Data points of the same colour represent data from the same donor.

*AKR1B1* and *SORD* transcripts were both found to be abundantly expressed in the hair matrix of control occipital scalp human HFs, notably within the proliferative zone below Auber's line [43](Figure 4A–C). Interestingly, *SORD* labelling was also seen to extend into the inner root sheath (IRS) layers (Figure 4C). Analysis showed that there was no significant change in the expression of *AKR1B1* and *SORD* in the hair matrix in AA versus HFs in control healthy scalp tissue, showing stable expression of these transcripts in AA versus healthy HFs (Figure 4B–D).

We also assessed SORD and AKR1B1 protein expression in healthy control, nonlesional AA and lesional AA scalp. AKR1B1 immunoreactivity was identified in the matrix, IRS, outer root sheath (ORS) and connective tissue sheath (CTS) (Figure 4E, Figure S3). Quantitative analysis of AKR1B1 protein expression revealed a significant increase in intensity in the lesional ORS and CTS compared to nonlesional and control. AKR1B1 expression was also significantly increased in the nonlesional CTS compared to the control (Figure 4F). A similar but nonsignificant trend was observed in the IRS (Figure S3). Matrix immunoreactivity was present in some samples but was qualitatively low compared to other compartments and therefore not analysed.

SORD protein expression was found primarily in the ORS, although staining was also observed in the dermal papilla (DP) and IRS in some cases (Figure 4G). However, quantitative analysis of SORD protein revealed that this was unchanged in AA, as there was no significant difference in expression between healthy control, nonlesional and lesional AA scalp HFs in the ORS (Figure 4H), or in the matrix or DP (Figure S3).

Finally, preliminary immunofluorescence staining of the fructose transporter, GLUT5, showed immunoreactivity within the IRS (Figure S3). Together, these data show the expression of polyol pathway enzymes required for endogenous fructose production in human HFs. Moreover, the specific increase in AKR1B1 protein expression in lesional AA may enable heightened local fructose production, thereby contributing to HF inflammation and endothelial activation, perhaps via UA generation or directly via oxidative stress [36].

## 4 Discussion

### 4.1 Linking Uric Acid and 13‐HODE to Oxidative Stress and Endothelial Activation in AA

Our work demonstrates that, like other comparable IMIDs [10, 11, 12], AA is characterised by metabolic changes that may disrupt hair growth and drive or amplify pathogenesis [15]. This is consistent with reported clinical associations of metabolic disorders; a cross‐sectional study of 33 000 patients found increased rates of metabolic disorders such as metabolic syndrome and diabetes mellitus in association with AA [44]. Similarly, a meta‐analysis of 102 studies also found increased rates of metabolic syndrome, though not diabetes mellitus, in AA [45].

Notably, we observed increased UA and decreased 13‐HODE in lesional AA biopsy sites, which are biomarkers associated with endothelial dysfunction [31, 33, 46]. In line with endothelial dysfunction in AA, which may permit enhanced lymphocyte extravasation [4] following IP collapse, we also show increased endothelial ICAM‐1 expression in AA scalp, even at nonlesional sites, and replicate this response in vitro with UA using HFOC. Interestingly, previous work has demonstrated that ICAM‐1 is broadly increased in lesional AA HFs, particularly within the ORS and dermal papilla [47]. It is possible that the endothelial activation demonstrated in our data may make up part of this broader increase. However, as ICAM‐1 expression is also known to increase in response to inflammation elsewhere (e.g., immune cells, epithelial cells) [48], further work is required to identify the primary contributors to broader ICAM‐1 increases in AA.

UA is generated via purine metabolism and increased as a byproduct of fructose metabolism [20, 49] and high levels are associated with oxidative stress, particularly in the context of cardiovascular damage [50]. It can promote endothelial damage via impaired nitric oxide release, HMGB1/RAGE signalling and the induction of oxidative stress [19], which may link UA to reports of oxidative stress and decreased antioxidants in AA [51].

UA and endothelial activation may serve as broader markers of hair loss disorders, supported by previous reports of elevated serum UA [52], microvascular regression and increased cardiovascular risk in androgenetic alopecia [53, 54]. This is further supported by our own data showing significantly increased serum UA in AA.

13‐HODE acts to reduce thrombogenicity and atherosclerotic risk [33]; therefore, its decrease at lesional AA sites could further contribute to endothelial activation or dysfunction. 13‐HODE is synthesised by ALOX‐15, which is highly expressed in AA [55]. As 13‐HODE levels are reduced, this could point to reduced substrate availability or a functional switch in ALOX15 towards the detoxification of lipid hydroperoxides during oxidative stress [56]. Interestingly, ALOX15 knockout mice show progressive hair loss and HF immune infiltration, reminiscent of AA [57].

### 4.2 A Role for the Polyol Pathway in AA‐Associated Endothelial Activation?

UA upregulates the polyol pathway, likely through oxidative stress‐induced NFAT5 activation and AKR1B1 activation [21]. Endogenous fructose production occurs in other tissues such as the liver [58], and the impact of UA‐polyol pathway interplay on liver disease and endothelial damage is well documented [21, 40, 41, 59, 60], and both UA and the polyol pathway are known to mediate activation of the NLRP3 inflammasome [61, 62]. In HFs, endogenous fructose may be utilised via GLUT5 and other fructose transporters to promote the Warburg effect to sustain hair growth [14, 63]. While our analysis showed no significant changes in *AKR1B1* and *SORD* transcripts or SORD protein expression, our finding showing a significant increase in AKR1B1 protein expression in AA scalp supports the hypothesis that the polyol pathway is dysregulated in AA HFs, potentially contributing to endothelial activation or dysfunction.

Additionally, the polyol pathway and purine degradation impact the availability of AMP and NADPH and are associated with oxidative stress and reduced AMPK activity [64, 65]. AMPK serves a critical role in protecting against endothelial damage [64, 66] and maintenance of Treg immunosuppressive capacity [67], as well as promoting hair growth [68, 69]. Therefore, polyol pathway dysregulation warrants further study as a potential contributor to weakened IP and/or endothelial activation in AA.

### 4.3 Alternate Potential Routes to Locally Increased UA Levels

Further investigation is required to identify the cause of locally altered UA levels between nonlesional and lesional regions. Systemic UA increases could be explained by dietary purine/fructose intake, with local changes resulting from differential sensitivity or import/export of UA in active AA lesions [70, 71].

Another area of speculation could be the altered skin microbiome in AA [72]. Alterations in the skin microbiome of psoriasis correlate with changes to plasma metabolome and UA levels, demonstrating the impact the skin microbiome can have on the metabolome [73]. Therefore, future work should consider how changes in the microbiome in AA might influence metabolic homeostasis.

As our exploratory metabolomics compared lesional with nonlesional scalp, cautious interpretation is required, where changes in UA levels relative to healthy control scalp remain unknown. This is important to bear in mind, as previous work has highlighted that nonlesional AA HFs show higher levels of pro‐inflammatory Vδ1+ T cells than healthy controls, and have CD4+ and CD8+ T cell levels that more closely resemble lesional AA rather than healthy control skin [74]. This may suggest that nonlesional AA scalp exists in an intermediary, potentially primed, state that exists on a disease spectrum between lesional scalp and healthy scalp. Indeed, our own CD31/ICAM‐1 endothelial activation stains show increased activation in nonlesional scalp compared to healthy controls, further supporting this concept.

### 4.4 Cardiovascular Risk in AA Patients

Increased markers of endothelial activation and dysfunction may indicate heightened cardiovascular risk, similar to comparable IMIDs like psoriasis and rheumatoid arthritis [75, 76], though whether AA patients demonstrate this increased risk has been a subject of debate. Some papers report a higher risk of acute myocardial infarction and stroke in AA patients [77, 78], along with markers of cardiovascular and atherosclerotic risk, and endothelial dysfunction [79, 80]. However, others find no such link [81] or associate increased risk only with severe AA in the form of AT and AU [82].

High UA levels have been linked to increased risk of heart failure [83] and increased circulating inflammatory cytokines [84], as well as being shown to act as an independent risk factor for hypertension [85] and being capable of inducing endothelial dysfunction in vitro [86]. As such, our findings of increased endothelial activation in AA scalp skin and increased serum UA levels (a common indicator of cardiovascular risk) support continuing research into AA patients' cardiovascular risk. Assessing endothelial dysfunction and cardiovascular risk in patients early in the disease course can allow clinicians to better respond to patients' needs, especially when considering data showing cardiovascular risk increases with disease duration [77].

## 5 Conclusion

Our data provides the first evidence of local metabolic changes in active scalp AA lesions. Metabolites such as UA and 13‐HODE, implicated in endothelial function and damage, are altered in AA scalp skin. CD31/ICAM‐1 immunofluorescence supported the presence of endothelial activation in AA scalp, and culture experiments highlight the capacity of UA and fructose to induce endothelial ICAM‐1 expression. Further research is warranted to assess the relationship between UA‐induced oxidative stress and endothelial dysfunction in AA, and whether targeting these UA levels directly or indirectly via pharmacological (e.g., xanthine oxidase inhibitors or aldose reductase inhibitors) or dietary interventions can restore microvascular function and promote hair regrowth in AA patients.

## 6 Limitations

The low sample size (*n* = 4) of the initial LC–MS tissue analysis limits the strength of the conclusions that can be drawn. Similarly, the lack of healthy control tissue in this analysis limits the strength of any comparison of UA changes between intrapatient tissue (lesional versus nonlesional) and interdonor (healthy versus AA) serum. Additionally, serum analysis was performed using samples that did not control for interindividual variability or dietary purine/fructose intake, so we are unable to assess the degree to which diet might influence these data.

## Author Contributions

**Madoc Dawson:** conceptualisation, methodology, validation, formal analysis, investigation, writing – original draft, visualisation. **Derek Pye:** investigation, data curation, writing – review and editing. **Rebecca Mahon:** methodology, investigation, writing – review and editing. **George Taylor:** methodology, formal analysis, investigation, resources, data curation, writing – review and editing. **Asim Shahmalak:** resources, writing – review and editing. **Bessam Farjo:** resources, writing – review and editing. **Nilofer Farjo:** resources, writing – review and editing. **Matthew Harries:** resources, data curation, writing – review and editing, funding acquisition. **Talveen S. Purba:** conceptualisation, writing – review and editing, supervision, project administration, funding acquisition.

## Funding

This work was supported by Alopecia UK (AUK2022_004) and the National Institute for Health and Care Research (NIHR203308).

## Disclosure

IRB Approval Status: West Midlands–South Birmingham REC (ref. [24]/NW/0044; IRAS No. 335918); North West–Haydock REC (ref. [19]/NW/0082; IRAS No. 260051).

Reprint Requests: Madoc Dawson.

## Consent

Consent for the use of hair follicle and scalp samples utilised in the production of this article was obtained by the authors and remains on file. No identifiable or recognisable material is presented in this article.

## Conflicts of Interest

The authors declare no conflicts of interest.

## Supporting information


**Figure S1:** (A) Representative immunofluorescence microscopy of CD31 and ICAM‐1 dual stains in the peribulbar region and papillary dermis of healthy control, nonlesional AA, and lesional AA scalp. Alternative pseudocoloured versions of each image are included beneath the accompanying image to improve legibility for those with RGB colour blindness. (B) Column scatter plots of dual‐positive CD31+/ICAM‐1+ cells as a percentage of all CD31+ cells in the peribulbar region and papillary dermis of control, nonlesional AA and lesional AA scalp (*n* = 5, 2–4 HFs per patient per condition) (one‐way ANOVA, Dunnett's multiple comparisons test, *p* = < 0.05). *p = < 0.05; **p = < 0.01; ***p = < 0.001; ****p = < 0.0001. Line at the median. Data points of the same colour represent data from the same HF donor. (C) Additional higher magnification (x20) representative immunofluorescence microscopy images of CD31 and ICAM‐1 dual stains in the peribulbar region of healthy control, nonlesional and lesional AA scalp. The white arrow indicates an example of co‐expression (yellow) of CD31 and ICAM‐1. Note—these images were taken with a different microscope and higher magnification and are not directly comparable to the images in (A) and (B).

**Figure S2:** (A) Representative immunofluorescence microscopy of CD31 and ICAM‐1 dual stains in human hair follicles cultured with uric acid (UA) for 72 h (control, 200 uM, 1000 uM). (B) Representative immunofluorescence microscopy of CD31 and ICAM‐1 dual stains in human hair follicles cultured with supplemental fructose or glucose for 72 h (control, 10 mM fructose/glucose, 40 mM fructose/glucose). All images are accompanied by an alternative pseudocoloured version to improve legibility for those with RGB colour blindness.

**Figure S3:** (A) Representative immunofluorescence microscopy of *AKR1B1* RNAScope in healthy control, nonlesional AA and lesional AA scalp. (B) Representative immunofluorescence microscopy of *SORD* RNAScope in healthy control, nonlesional AA, and lesional AA scalp. (C) Representative immunofluorescence microscopy of AKR1B1 immunohistochemistry in healthy control, nonlesional AA and lesional AA scalp. (D) Representative immunofluorescence microscopy of SORD immunohistochemistry in healthy control nonlesional AA and lesional AA scalp. (E) Positive control representative immunofluorescence microscopy of AKR1B1 immunohistochemistry in human liver. (F) Positive control representative immunofluorescence microscopy of SORD immunohistochemistry in human liver. (G) Representative immunofluorescence microscopy of GLUT5 in healthy control hair follicles. (H) Column scatter plots of nonsignificant differences in AKR1B1 inner root sheath (IRS) and SORD bulbar matrix and dermal papilla fluorescence intensity (FI) in healthy control, nonlesional AA and lesional AA scalp (control *n* = 4, NL/L *n* = 5) (one‐way ANOVA, Tukey's multiple comparisons test, no significant differences, *p* = > 0.05) (all conditions compared to all other conditions; nonsignificant results not shown on graph).

**Table S1:** A list of all metabolites in the liquid chromatography mass spectrometry data set found by one‐way ANOVA to be significantly altered between lesional and nonlesional AA scalp.

## Data Availability

The data that support the findings of this study are available from the corresponding author upon reasonable request.
