# Supplementary figures and images for: Metabolic and Vascular Inflammation in Alopecia Areata: Linking Uric Acid, Lipid Imbalance and ICAM‐1 Upregulation
Source: Exp Dermatol. 2025 Dec 12;34(12):e70186. doi: 10.1111/exd.70186 (PMC12700771; doi:10.1111/exd.70186)

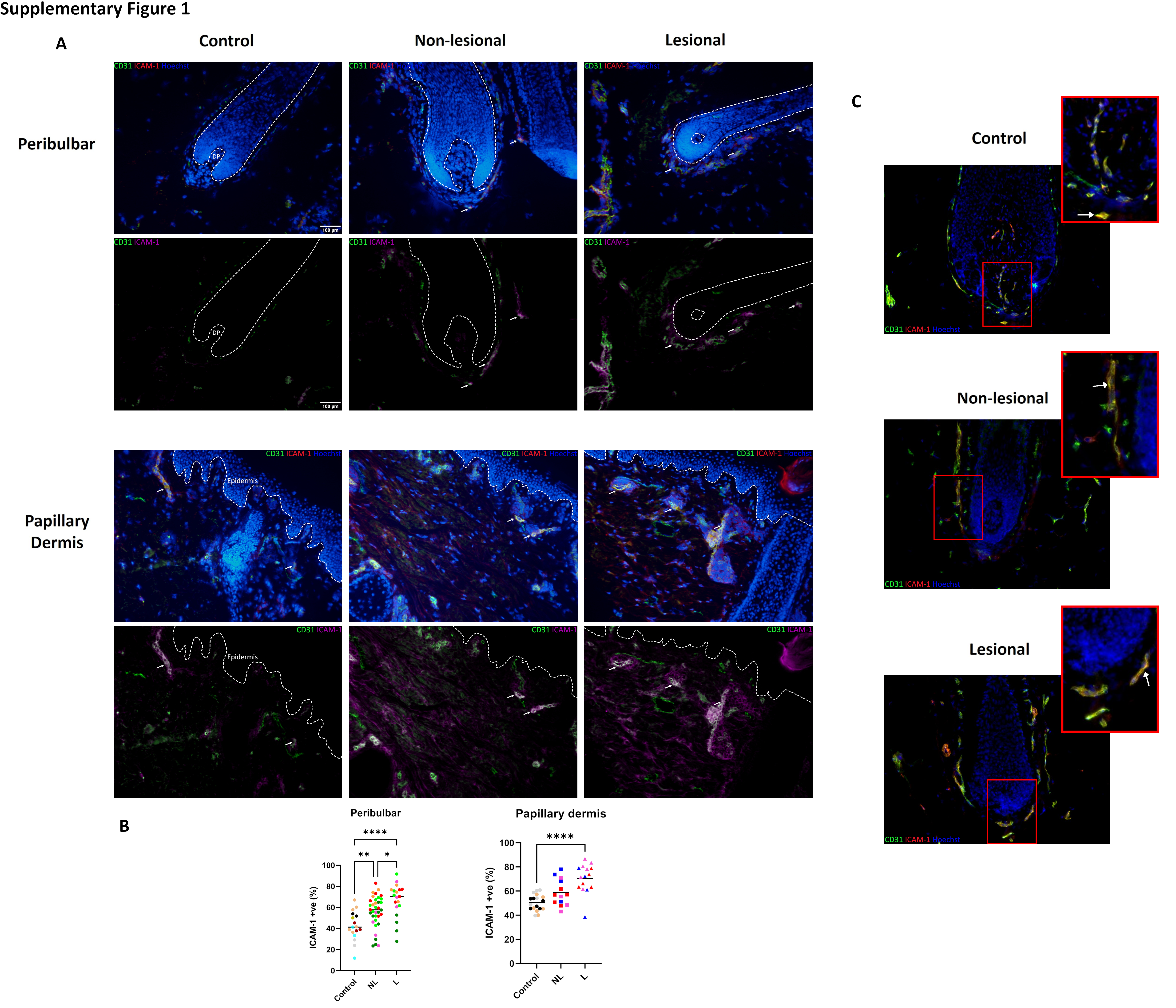

Supplement: Supplementary file 1 — Figure S1: (A) Representative immunofluorescence microscopy of CD31 and ICAM‐1 dual stains in the peribulbar region and papillary dermis of healthy control, nonlesional AA, and lesional AA scalp. Alternative pseudocoloured versions of each image are included beneath the accompanying image to improve legibility for those with RGB colour blindness. (B) Column scatter plots of dual‐positive CD31+/ICAM‐1+ cells as a percentage of all CD31+ cells in the peribulbar region and papillary dermis of control, nonlesional AA and lesional AA scalp (n = 5, 2–4 HFs per patient per condition) (one‐way ANOVA, Dunnett's multiple comparisons test, p = < 0.05). *p = < 0.05; **p = < 0.01; ***p = < 0.001; ****p = < 0.0001. Line at the median. Data points of the same colour represent data from the same HF donor. (C) Additional higher magnification (x20) representative immunofluorescence microscopy images of CD31 and ICAM‐1 dual stains in the peribulbar region of healthy control, nonlesional and lesional AA scalp. The white arrow indicates an example of co‐expression (yellow) of CD31 and ICAM‐1. Note—these images were taken with a different microscope and higher magnification and are not directly comparable to the images in (A) and (B). [file EXD-34-e70186-s004.png]

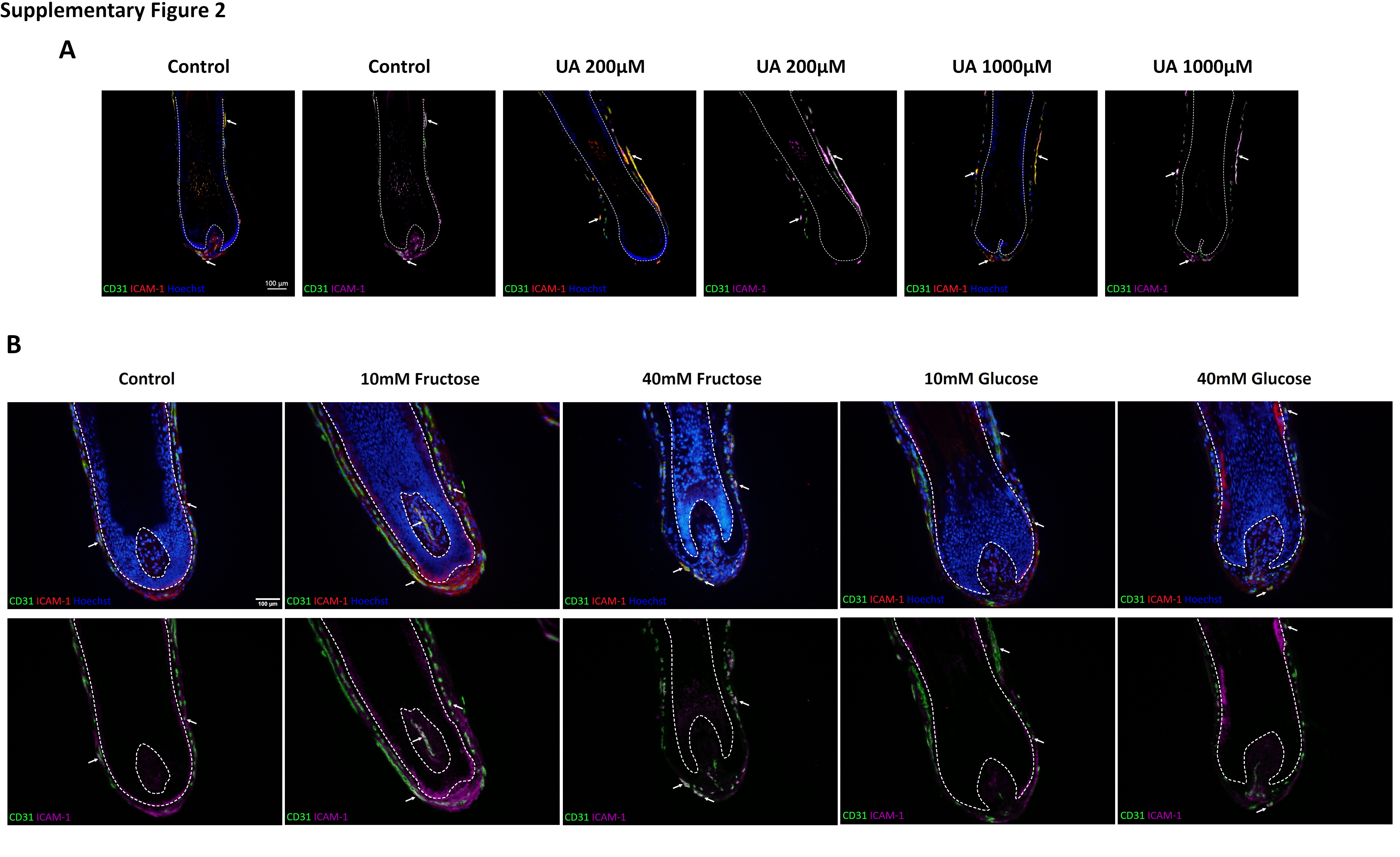

Supplement: Supplementary file 2 — Figure S2: (A) Representative immunofluorescence microscopy of CD31 and ICAM‐1 dual stains in human hair follicles cultured with uric acid (UA) for 72 h (control, 200 uM, 1000 uM). (B) Representative immunofluorescence microscopy of CD31 and ICAM‐1 dual stains in human hair follicles cultured with supplemental fructose or glucose for 72 h (control, 10 mM fructose/glucose, 40 mM fructose/glucose). All images are accompanied by an alternative pseudocoloured version to improve legibility for those with RGB colour blindness. [file EXD-34-e70186-s001.png]
