# Supplementary material for: Metabolic and Vascular Inflammation in Alopecia Areata: Linking Uric Acid, Lipid Imbalance and ICAM‐1 Upregulation
Source: Exp Dermatol. 2025 Dec 12;34(12):e70186. doi: 10.1111/exd.70186 (PMC12700771; doi:10.1111/exd.70186)
Supplement: Supplementary file 4 — Table S1: A list of all metabolites in the liquid chromatography mass spectrometry data set found by one‐way ANOVA to be significantly altered between lesional and nonlesional AA scalp. [file EXD-34-e70186-s002.docx]

| Metabolite | Increased or decreased in lesional | P-value |
| --- | --- | --- |
| DL-2-Methylbutyric acid | Decreased | 0.000184 |
| Theaflavine | Decreased | 0.00499 |
| Fraxetin | Increased | 0.006876 |
| 19-Norandrosterone | Decreased | 0.01007 |
| Hexose sugar | Increased | 0.011674 |
| Arg-Lys | Decreased | 0.014197 |
| Trp-Met(O) | Decreased | 0.018135 |
| Gln-Met(O)-Lys | Decreased | 0.02 |
| (-)-Epinephrine | Increased | 0.020324 |
| 11.beta.,17.alpha.,21-Trihydroxy-5.alpha.-pregnane-3,20-dione | Decreased | 0.020945 |
| Glycerol 1-myristate | Decreased | 0.021024 |
| Monolaurin | Decreased | 0.021318 |
| L-Aspartic acid | Increased | 0.023615 |
| Trp-Phe-Lys | Increased | 0.025386 |
| Ala-Val-Arg | Decreased | 0.028065 |
| 1-Hexadecanoyl-sn-glycerol | Decreased | 0.0293 |
| 10-Hendecenoic acid | Decreased | 0.030708 |
| 5-Hydroxyisovanillic acid | Increased | 0.033269 |
| 6''-O-Acetylglycitin | Increased | 0.034653 |
| 12(S)-Hydroxy-16-heptadecynoic acid | Decreased | 0.034701 |
| His-Ser-Arg | Increased | 0.034715 |
| 1,2-Dioctanoyl-sn-glycerol | Decreased | 0.03859 |
| Asp-Tyr | Increased | 0.039369 |
| Glycocholic acid | Decreased | 0.039752 |
| Cys-Cys-Lys | Decreased | 0.040835 |
| 6.alpha.-Mannobiose | Increased | 0.04198 |
| Trp-His | Increased | 0.0437 |
| Malonic acid | Increased | 0.044729 |
| 13-HODE | Decreased | 0.045598 |
| 1-(10Z-Heptadecenoyl)-sn-glycero-3-phosphocholine | Decreased | 0.047483 |
| D-Glucoheptose | Increased | 0.047623 |
| Uric acid | Increased | 0.047976 |

**Supplementary Table 1**

A list of all metabolites in the liquid chromatography mass spectrometry data set found by one-way ANOVA to be significantly altered between lesional and non-lesional AA scalp.
